# If SARS-CoV-2 vaccination is blamed for Parsonage-Turner syndrome, neurosurgical neurolysis is not indicated

**DOI:** 10.51866/lte.46l

**Published:** 2024-05-23

**Authors:** Fulvio A Scorza, Josef Finsterer

**Affiliations:** 1 MD, PhD, Neurology and Neurophysiology Center , Postfach 20, Vienna, Austria. Email: fifigs1@yahoo.de; 2 MD, Federal University of Sao Paolo, Sao Paolo, Brazil.

**Keywords:** Plexitis, SARS-CoV-2, Vaccination, Side effect, Muscle weakness

**Dear editor,**

We read with interest the article by Yeoh and Ramdzan describing the case of a 50-year-old man who was diagnosed with Parsonage-Turner syndrome (PTS) after vaccination with a booster dose of an mRNA-based SARS-CoV-2 vaccine.^1^ Symptoms started 5 days after vaccination, but the patient did not come to the hospital until 9 weeks after symptom onset. The patient benefitted from neurosurgical neurolysis of C5, C6 and the upper trunk as well as low-level laser therapy. The study offers valuable information but has limitations that should be discussed.

The main limitation of the study is that alternative causes of PTS were not completely ruled out. The patient underwent neither cerebrospinal fluid examination nor magnetic resonance imaging with a contrast medium to rule out enhancement of the radices of C6-8 or a tumour. An HbAlc or oral glucose tolerance test was not performed to rule out diabetes. Further, the study did not mention whether the patient was alcoholic. No virus panel test was conducted, and no PCR test was performed for SARS-CoV-2. Despite vaccination, SARS-CoV-2 infection is possible, which has been shown to be complicated by PTS.^2^ In the study, there was no mention of whether the patient developed a traumatic plexus lesion before the onset of the clinical presentation. It was also unclear whether the patient had a previous viral, parasitic or bacterial infection or a rheumatologic disease and received immunisation with a vaccine other than a SARS-CoV-2 vaccine. Moreover, a point mutation in the *SEPT9* gene was not sufficiently ruled out as the cause of PTS. Generally, *SEPT9* gene mutations can occur in 50% of patients with hereditary neuralgic amyotrophy.^3^ In addition, multifocal motor neuropathy (MMN) must be ruled out. Since the levels of GM1 antibodies are elevated in most cases of MMN,^4^ it is essential to determine whether the levels were elevated in the patient.

It was unclear why the patient underwent neurolysis. The treatment of PTS rarely requires surgery. There is definitively no indication for surgery if PTS is due to an immunological mechanism after SARS-CoV-2 vaccination. However, the significant reduction in pain after surgery suggested that PTS was not due to vaccination but to an aetiology causing brachial plexus compression. Whether there was evidence of compression of the C6 and C7 roots and the upper trunk that indicated neurolysis was not reported. The outcomes of neurolysis of C6 and C7 suggested that the patient had radiculopathy rather than plexopathy.

As shown in Figure 2 in the study, the patient had gynaecomastia. It was not mentioned whether there was evidence of hormonal imbalance, obesity, liver cirrhosis or a benign tumour. The cause of gynaecomastia and the presence or absence of a causal relationship with PTS should be clarified.

Although the study provides valuable information, it has limitations that raise doubts about the results and their interpretation. Addressing these issues could strengthen the conclusions and improve the status of the study. Before SARS-CoV-2 vaccination is blamed for PTS, alternative aetiologies must be ruled out.

## References

[1] Yeoh ZY, Ramdzan SN (2023). Parsonage-Turner syndrome: a case report of a rare side effect of COVID-19 booster vaccination.. Malays Fam Physician..

[2] Cornea A, Lata I, Simu M, Rosca EC (2023). Parsonage-Turner syndrome following SARS-CoV-2 infection: a systematic review.. Biomedicines..

[3] Neubauer K, Boeckelmann D, Koehler U (2019). Hereditary neuralgic amyotrophy in childhood caused by duplication within the SEPT9 gene: a family study.. Cytoskeleton (Hoboken)..

[4] Hameed S, Cascella M (2023). StatPearls..

